# Correcting Scoliosis in Rett Syndrome

**DOI:** 10.7759/cureus.15411

**Published:** 2021-06-03

**Authors:** Brett Rocos, Reinhard Zeller

**Affiliations:** 1 Department of Orthopaedic Surgery, Hospital for Sick Children, Toronto, CAN

**Keywords:** rett, scoliosis, syndromic scoliosis, neuromuscular scoliosis

## Abstract

Objectives

Rett syndrome is a rare disorder characterised by severe scoliosis in 80% of cases. In this retrospective case series, we analysed the radiographic, clinical, and functional outcomes of consecutive patients treated for scoliosis associated with Rett syndrome. We sought to understand the results of the treatment of scoliosis in Rett syndrome and evaluate the need to fuse to the pelvis.

Methods

A retrospective case series was used to analyse the radiographic, clinical, and functional outcomes of consecutive patients treated for Rett syndrome scoliosis between the ages of 10 and 8 years in a single tertiary paediatric spinal unit. Cases were identified through departmental and neurophysiological records, and patients were excluded if the diagnosis of Rett syndrome was not confirmed.

Results

Seven eligible cases were identified. At presentation, the mean coronal Cobb angle was 90.9°, mean sagittal Cobb 72.0°, and pelvic obliquity 24.5°. The mean post-operative improvement in coronal Cobb was 53.2° and pelvic obliquity reduced to 5.8°. These did not change during a mean follow up of 3.5 years. None showed any post-operative complications. Three patients with a mean 16.1° pelvic obliquity underwent a fusion to L5. The postoperative result in those cases remained stable at 3.5 years mean follow-up and full skeletal maturity.

Conclusion

Our data suggests that with modern technology, severe curves can be safely treated. Fusion to the pelvis is not necessary in patients with mild, flexible pelvic obliquity.

## Introduction

Rett syndrome is a rare, neurodegenerative condition which affects young patients, predominantly females, in the first two years of life [1-3]. Investigations show that it is most commonly caused by a mutation in methyl-CpG-binding protein 2 (MECP2), though others have been implicated [1,4-6]. The features of Rett syndrome includes hypotonia, weakness, gross motor disturbance, and typical writhing hand movements [7,8]. As an affected child develops, the orthopaedic manifestations of the disease become more apparent, including lower extremity contractures, coxa vara, and scoliosis in up to 83% of patients [1,9-15]. Common to many of the neuromuscular causes of spinal deformity, a long C-shaped scoliosis is the usual pattern, however, in the case of Rett syndrome, rapid progression in the second decade often occurs [13,16].

The consequence of scoliosis is reduced mobility, reduced sitting balance, and in some severe cases, reduced pulmonary function [17]. Evidence suggests that to limit these, the scoliosis should be treated when the coronal deformity reaches 40° [15,16,18]. It has been suggested that patients with Rett-associated scoliosis should be treated with fusion to the pelvis in order to prevent subsequent progression of deformity correct pelvic obliquity [19]. However, with the additional surgical morbidity attached to fusion to the pelvis, it is sensible to avoid this additional insult where possible, with the potential benefit of preserving ambulation in some cases [5].

In this case series, we sought to describe the severity of spinal and spinopelvic deformity observed in Rett syndrome, the clinical and radiological outcomes of surgery, and compare outcomes between patients who were fused to either the pelvis or L5.

## Materials and methods

Local ethical board consent was granted. All patients aged between 10 and 18 years with a diagnosis of Rett syndrome who underwent posterior spinal fusion for scoliosis were identified using departmental records. The clinical records and digitally stored radiographs (Centricity Enterprise Web V3.0, GE Medical Systems, Arlington Heights, USA) of each patient were reviewed. The immediate post-operative radiograph and the radiograph taken closest to one-year follow up were assessed to evaluate the correction achieved. The most recent follow up radiograph was then compared with the post-operative radiograph to evaluate for any change in spinal alignment. Clinical records were examined to evaluate for the pre- and post-operative mobility, intraoperative neuromonitoring events, surgical complications, or revision procedures.

Data were recorded in Microsoft Excel v 16.32 (Microsoft, Redmont, WA, USA) and analysed using Stata v14 (StataCorp, College Station, TX, USA). Descriptive statistics were used to analyse the radiographic parameters and a two-tailed paired t-test used to assess for changes in curve magnitude in the follow-up period. Categorical data were described using free text.

## Results

Seven female patients met the inclusion criteria with a mean age at surgery of 14.1 years (range 11.0- 17.7). None had undergone any previous hip or spine surgery. Three patients were able to take at least one step and transfer from chair to chair prior to surgery. All showed a neuromuscular pattern to their curve, with a mean coronal Cobb angle of 90.9° (95% CI 76.0- 105.9°), kyphosis of 72.0° (95% CI 50.7- 93.3°) and pelvic obliquity of 24.5° (95% CI 11.3- 37.8°) (Table 1). Mean follow up was 3.5 years (± 1.65).

**Table 1:** The demographics and pre-operative radiographic measurements of the cohort

Patient No	Age at surgery (yrs)	Coronal Cobb (°)	Kyphosis (°)	Pelvic obliquity (°)	Apex of deformity
1	17.7	120.0	68.5	45.0	L2
2	16.7	89.0	72.5	14.5	T12
3	13.2	87.3	69.9	21.7	L1
4	11.0	104.8	100.7	38.1	T10
5	15.6	82.7	28.5	7.3	T11
6	11.0	79.5	70.7	12.1	T11
7	13.7	73.2	93.1	32.8	L2

Four patients were fused from T2 to the pelvis and three to L5 with facetectomy at each level. All underwent posterior instrumented fusion without osteotomy or anterior release. Six had tibial autograft applied to the spine, and two additional iliac crest autograft. All patients were treated with a hybrid construct. The mean length of inpatient stay was 10 days (95%CI 5.6- 15.0 days).

Four patients showed changes in intraoperative motor evoked potentials (MEPs), each treated with release of traction, elevation of mean arterial pressure and in a single case the temporary release of the correction. No intraoperative complications were encountered, and none showed any changes from their preoperative neurological status as a result of surgery when examined post operatively. The post-operative mean coronal Cobb was 37.7° (95% CI 27.9- 47.6°), a mean improvement of 53.2° (95% CI 40.5- 65.9, p< 0.01) (Table 2).

**Table 2:** The changes in the coronal deformity and ambulation status of each patient

Patient no	Ambulating pre op	Pre-operative Coronal Cobb (°)	Pelvic obliquity (°)	Fused to pelvis	Post-operative coronal Cobb (°)	Post-operative pelvic obliquity	Post-operative ambulation
1	N	120.0	45.0	Y	44.7	2.1	N
2	N	89.0	14.5	N	61.7	10.9	Y
3	Y	87.3	21.7	N	46.9	4.4	Y
4	N	104.8	38.1	Y	40.2	4.7	N
5	Y	82.7	7.3	Y	20.5	10.2	Y
6	Y	79.5	12.1	N	43.5	1	Y
7	N	73.2	32.8	Y	42.3	7	N

Kyphosis was improved by 35.4° (95% CI 13.1- 57.7, p < 0.01). Pelvic obliquity was improved to a mean 5.8° (Table 2). The spinal parameters were unchanged for the duration of follow up regardless of whether the pelvis was included in the construct or not. None of the patients showed any post-operative complications, and all survived to most recent follow up. No patient who was fused to the pelvis showed any change in their ability to ambulate following surgery.

The three patients not fused to the pelvis presented with a moderate flexible pelvic obliquity preoperatively. The residual 5.8° pelvic obliquity remained unchanged over a mean follow up of 3.5 years and full skeletal maturity at last follow-up (average age 17 years). A single patient of the three showed an improvement in their postoperative mobility (Table 2).

## Discussion

**Figure 1:**
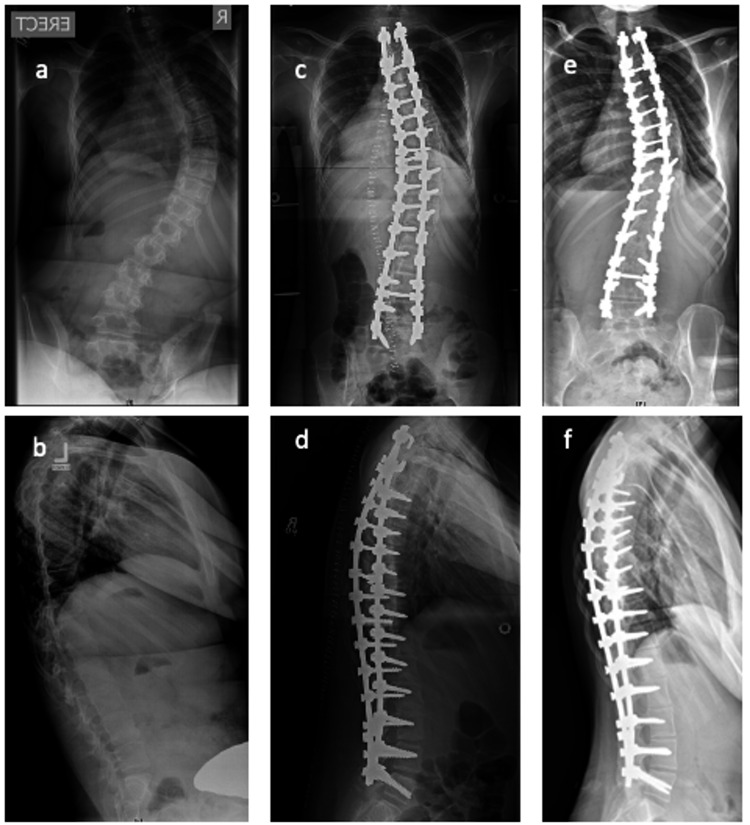
Plain radiographs of a 11-year-old female patient a) AP and b) lateral plain radiograph of an 11-year-old female patient with a coronal angular deformity of 79.5◦ and kyphosis of 70.7◦ and pelvic obliquity of 12.1◦. c) AP and d) lateral post-operative radiographs showing posterior instrumented fusion of T2 to L5 with a coronal angular deformity of 23.1◦, kyphosis of 43.5◦ and pelvic obliquity of 1◦. This correction is maintained at five years in e) AP and f) lateral radiographs.

These data suggest that, in selected cases, it may be possible to avoid fusion to the pelvis and prevent further deformity despite the progressive nature of the disease. Furthermore, it suggests that the incidence of postoperative complications is low when posterior fusion with tibial graft is used. A satisfactory radiological result is achieved without anterior release in all cases, and no incidence of neurological compromise is recorded (Figure 1).

It would be reasonable to recommend that patients with fixed pelvic obliquity, a history of hip surgery, and a declining neurologic status should undergo fusion to the pelvis to ensure a stable sitting posture. Although these data are unable to identify criteria which guarantee a successful outcome after a posterior spinal fusion not extending to the pelvis, it could be hypothesised that strategy is reasonable in patients who retain some control over the lumbosacral junction (for example, controlled swaying of the trunk) with symmetrical hip range of motion and some retention of motor control. In this series, patients were fused to the pelvis when there was a preceding rapid decline in neurological function with a lethargic presentation and uncontrolled, treatment-resistant daily seizures.

The clinical significance of these results is in preventing surgical morbidity. By avoiding fusion to the pelvis in almost half of the cases, we have shown that the overall outcome is satisfactory, and suggested that this strategy will aid in preserving a patient’s ability to independently mobilise. Furthermore, these findings provide evidence that tibial grafting is useful in achieving fusion in these complex cases, as there is no incidence of loose hardware or revision for pseudarthrosis seen in this series.

Given that the evidence supports tackling spine deformity in paediatric patients when it reaches 40°, these patients in this cohort could reasonably be defined as being at the severe end of the spectrum [3,15,16]. This is supported by Riise et al. and Koop et al., who both identify severe scoliosis in Rett syndrome as one with a long curve which the pelvis becomes oblique, and Killian et al. who showed that severe scoliosis in Rett syndrome correlated with a reduced ability to ambulate and use one’s hands [13,19,20]. The literature does not contain any studies which evaluate the corrections possible in Rett syndrome. This may be because of its rarity, or the technical challenges which have historically been present in managing these patients, the perioperative morbidity, and difficulty in monitoring their neurological status during the procedures [21,22]. Nonetheless, we have shown that a safe correction can be achieved and that modern techniques in neuromonitoring can facilitate its accomplishment.

Alongside the prevention of deterioration, patients and their families derive benefit from surgery in severe scoliosis associated with Rett syndrome. Kerr et al. showed that 84% of families thought that surgery had a positive effect on the family unit [15]. In 2016, Downs et al. also showed that surgery appears to lengthen life in this patient group, particularly in reducing the risk of respiratory tract infection, though in contrast. Pisano et al. report a patient who did not survive extension of fusion to the pelvis, and Gabos et al. showed a 63% rate of respiratory and 37% gastrointestinal complication rate in these patients [17,23,24].

This study is limited by the small number of patients treated for Rett syndrome, and the challenges in consistent radiographic technique in non-ambulatory patients. Although we have seen no loss to follow up, extending the follow up period into adulthood would be useful in understanding how mobility changes following surgery in later life.

## Conclusions

Scoliosis is the most common orthopaedic manifestation of Rett syndrome, and is one that should be considered for surgical treatment early in its development. Our data suggests that with modern technology, severe curves can be safely treated, that fusion to the pelvis is not always necessary, and in some selected cases, a fusion to L5 is sufficient and may be important in preserving patient mobility post operatively.
